# The influence of jaw-muscle fibre-type phenotypes on estimating maximum muscle and bite forces in primates

**DOI:** 10.1098/rsfs.2021.0009

**Published:** 2021-08-13

**Authors:** Megan Holmes, Andrea B. Taylor

**Affiliations:** ^1^ Department of Family Medicine and Community Health, Duke University School of Medicine, Durham, NC, USA; ^2^ Department of Basic Science, Touro University, Vallejo, CA, USA

**Keywords:** myosin heavy chain, maximum isometric tension, hybrid fibres, masseter, temporalis, muscle force

## Abstract

Numerous anthropological studies have been aimed at estimating jaw-adductor muscle forces, which, in turn, are used to estimate bite force. While primate jaw adductors show considerable intra- and intermuscular heterogeneity in fibre types, studies generally model jaw-muscle forces by treating the jaw adductors as either homogeneously slow or homogeneously fast muscles. Here, we provide a novel extension of such studies by integrating fibre architecture, fibre types and fibre-specific tensions to estimate maximum muscle forces in the masseter and temporalis of five anthropoid primates: *Sapajus apella* (*N* = 3), *Cercocebus atys* (*N* = 4), *Macaca fascicularis* (*N* = 3), *Gorilla gorilla* (*N* = 1) and *Pan troglodytes* (*N* = 2). We calculated maximum muscle forces by proportionally adjusting muscle physiological cross-sectional areas by their fibre types and associated specific tensions. Our results show that the jaw adductors of our sample ubiquitously express MHC α-cardiac, which has low specific tension, and hybrid fibres. We find that treating the jaw adductors as either homogeneously slow or fast muscles potentially overestimates average maximum muscle forces by as much as approximately 44%. Including fibre types and their specific tensions is thus likely to improve jaw-muscle and bite force estimates in primates.

## 1. Introduction

Considerable effort in anthropology has been directed at modelling feeding mechanics in extant primates with the goal of gaining insights into feeding-system adaptations in extant and extinct taxa. Research linking feeding-system design and function has relied heavily on biomechanical models based fundamentally on engineering principles. For example, from decades of *in vivo* experimental studies of how the mandible is strained during chewing, incision and isometric biting (e.g. [1–3]), a large body of literature has emerged applying beam theory to hypothesize how the mandibular corpus should be expected to model, remodel and adapt to withstand various loads during feeding, and to infer feeding behaviour and diet from mandibular morphology [4–9]. Additionally, the primate masticatory complex is often treated as a lever system (e.g. [10–12]), which, in conjunction with estimates of jaw-muscle force, have been used to model and estimate bite force capabilities (e.g. [13–18]). More recently, finite-element analysis (FEA) has become widely employed in biological anthropology to characterize the stress and strain patterns of the craniofacial complex under a variety of loading conditions (e.g. [19–22]) and to test hypotheses of craniofacial function and feeding adaptations (e.g. [23–26]).

All of these approaches are based on mechanical engineering principles and involve the application of a variety of input parameters. For instance, in FEA models of stress and strain patterns associated with feeding, bone tissue and bone material properties must be assigned, as well as applied muscle force vectors and muscle forces [24,25,27–29]. Similarly, bite force calculations require the input of muscle forces, often estimated from the anatomical (ACSA) or physiological cross-sectional area (PCSA) (e.g. [15,30–34]; for a review of methods for estimating PCSA see [35]). The validity of these models is in part dependent on the accuracy of the input parameters.

A focus of many studies of craniofacial structure and function has been modelling bite forces. This is because bite force is an important performance variable related to feeding (e.g. [36,37]) as well as non-feeding behaviours such as aggressive biting [38]. Bite force capacity is one of a number of factors that influence an animal's feeding strategies (e.g. seed predation, frugivory, folivory) and dietary range (e.g. accessing items across a range of material and geometric properties), and plays a crucial role in an animal's ability to efficiently ingest and break down food [39–41].

Bite force is modulated by a number of factors, including leverage (i.e. moment arms of the chewing muscles and bite points) and the force-generating capacity of the chewing muscles. Two important properties that influence muscle force are a muscle's PCSA and the physiological properties of muscle fibre types (fibre phenotype). Over the past 25 years, there has been a sizeable increase in static architectural estimates of ACSAs and PCSAs of the chewing muscles of strepsirrhine [30,33,42–46] and anthropoid [30–32,34,47–53] primates. This work has facilitated combining architectural estimates of jaw-adductor muscle force with muscle leverage to estimate bite force in extant [15,33,54,55] and, though a more formidable task, extinct human and non-human primates (e.g. [16,46,54,56]).

To convert muscle force estimates to bite force requires estimating the maximum isometric muscle force of the jaw adductors, i.e. the masseter, temporalis and medial pterygoid muscles. This is achieved by multiplying muscle ACSAs or PCSAs by a specific tension, i.e. force/area [57]. To date, however, studies estimating muscle force in primates, and converting muscle force to bite force, have assigned a specific tension value to muscle force estimates derived using whole muscles, and even muscle groups (e.g. [58–60]). This is despite considerable intra- and intermuscular heterogeneity of muscle fibre types and their contractile properties, including specific tensions (see table 1 and references therein; see Close [77] for an early review).

**Table 1.** Range of specific tension values reported in the literature. Both the muscles and muscle level sampled are reported.^a^

mammal(s)	muscle(s)	muscle level sampled	specific tension reported (N cm^−2^)	author
cat	gastrocnemius, soleus	whole muscle	22.5	Spector *et al*. [59]
cat	gastrocnemius, soleus	single fibre	10.3–43.8	Lucas *et al*. [61]
cat	flexor digitorum longus	single motor unit	5.9–34.3	Dum *et al*. [62]
cat	tibialis anterior	single motor unit	16.8–27.5	Bodine *et al*. [63]
cat, dog	diaphragm, longissimus dorsi, masseter, semimembranosus, soleus, temporalis, tibialis anterior	single fibre	10.2–38	Toniolo *et al*.^b^ [64]
chimpanzee	gastrocnemius, vastus lateralis	single fibre	9.6–15	O'Neill *et al*. [65]
cow	diaphragm, longissimus dorsi, masseter	single fibre	5–11.3	Toniolo *et al*. [66]
dog	longissimus dorsi, semimembranosus, extraocular, laryngeal, temporalis	single fibre	10–13	Toniolo *et al*.^b^ [67]
human	vastus lateralis	single fibre	6.2–22.2	Gilliver *et al*. [68]
human	soleus, vastus lateralis	single fibre	8–21	Larsson & Moss [69]
human	latissimus dorsi	single fibre	11.6–16.4	Paoli *et al*. [70]
human	vastus lateralis	single fibre	4.37–6.47	Bottinelli *et al*. [71]
mouse	gastrocnemius, soleus, tibialis anterior, vastus lateralis	single fibre	22.4–37.7	Andruchov *et al*. [72]
mouse, rat, rabbit, sheep, cow	extensor digitorum longus, soleus	single fibre	6–24.8	Seow & Ford [73]
mouse, rabbit, rat, human	extensor digitorum longus, gastrocnemius	single fibre	4–7.5	Pellegrino *et al*. [74]
rabbit	extraocular	single fibre	14–46	Lynch *et al*.^b^ [75]
rat	extensor digitorum longus, soleus, plantaris	single fibre	21.1–43.9	Bottinelli *et al*. [76]

^a^The ranges reported for each study reflect variation across muscles, fibre types within a muscle and/or experiments under different conditions.

^b^These values were not reported directly in the text. They were determined to the nearest approximation from graphic data presented by the author.

Here, we present a novel extension of these studies by combining jaw-adductor muscle force estimates from PCSAs with muscle fibre types quantified from the same muscles. We assign specific tension values (*P*_o_) based on the percentage cross-sectional area (%CSA) of fibres expressing a particular fibre type by taking advantage of recent empirically derived *P*_o_ values for the major myosin heavy chain (MHC) proteins that contribute to the fibre types found in mammalian jaw muscles. We compare our fibre-type adjusted muscle force estimates with those obtained assuming either homogeneously slow or homogeneously fast fibre phenotypes. We show that jaw-muscle force estimates based on the presumption of homogeneous fibre types assigned a single specific tension potentially overestimate average maximum muscle forces by as much as 44% and 36% for the masseter and temporalis muscles, respectively. These data add to the longstanding work of anthroengineering by providing a method for potentially fine-tuning jaw-muscle and bite force estimates in primates.

### 1.1. Estimating muscle force

Muscle function is largely dependent on two intrinsic properties of muscle: muscle fibre architecture [78] and the composition and distribution of fibre types within a muscle [76,79,80]. Muscle fibre architecture represents the internal organization of a muscle and the orientation of fibres relative to the muscle's force-generating capacity [78]. PCSA is proportional to a muscle's maximum force-generating capacity [57], while the expression and distribution of the various MHC isoforms within a muscle influence the contractile properties of muscle fibres, including speed of shortening, isometric tension and tension cost [81]. Combining a muscle's PCSA with its specific tension allows for an estimate of a muscle's maximum tetanic tension.

Numerous studies have combined architectural estimates of muscle force with specific tension values to estimate maximum tetanic tension for limb (e.g. [57,82,83]) and jaw musculature (e.g. [16,51]). A wide range of specific tension values, from as low as 4 N cm^−2^ [74] to as high as 46 N cm^−2^ [75] (table 1), have been reported for skeletal muscles and attributed to fibre type. Studies have generally assigned a single specific tension value to whole muscles, or to muscle groups. However, there is ample evidence from single-fibre studies that tension values are lower in slow (type 1) as compared with fast (type 2) fibres, in many cases significantly so (e.g. [62–67,70,71,74–76]).

**Figure 1.**
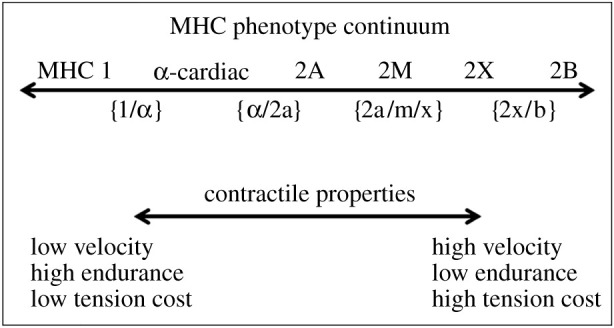
Fibre phenotype continuum illustrating the six main myosin heavy chain isoforms observed in mammalian jaw muscles, each operating at different capacities along the continuum. Hybrid fibres are denoted with brackets below the arrow but may occur in any combination. Maximum unloaded shortening velocity and tension cost increases from left to right while fatigue resistance decreases left to right.

Fibre-specific tensions have the potential to significantly influence muscle and, by extension, bite force estimates. One reason is that mammalian chewing muscles express a greater variety of MHC isoforms than limb muscles [84]; these isoforms add considerably to the range of contractile properties expressed in the chewing muscles. Another reason is that mammalian chewing muscles show a large degree of heterogeneity in fibre-type distribution, both within and between muscles (e.g. [85,86]). Lastly, mammalian chewing muscles express large quantities of hybrid fibres, i.e. fibres that express more than one MHC isoform along their length (see reviews by [87,88]). Notably, anthropoid primate chewing muscles display an abundance of hybrid fibres [89,90]. Fibres expressing multiple MHC isoforms are reported to have contractile properties intermediate between ‘nearest neighbour’ pure MHC isoforms [91] (figure 1). For the chewing muscles, in particular, these factors collectively suggest that applying a single specific tension value to whole muscles is likely to over- or underestimate maximum muscle tension and, thus, bite forces.

### 1.2. Jaw-muscle fibre phenotypes

A muscle's fibre phenotype is the quantitative expression of MHC isoforms within that muscle. The contractile properties of these isoforms can be expressed along a continuum, from slower contracting, fatigue resistant (type 1: MHC-1, MHC α-cardiac) to fast contracting, fatigable (type 2: MHC-2A, MHC-M, MHC-2X; figure 1). MHC-1 and MHC-2 (MHC-2A and MHC-2X) isoforms, common to the limb muscles, are expressed in mammalian jaw-closing muscles, including those of primates [86,92–94]. Additional major MHC isoforms are expressed in the chewing muscles, but not the limb muscles. These include MHC α-cardiac [95] and MHC-M (fast-type 2 or masticatory myosin) [85]. MHC α-cardiac has been reported in a variety of mammals (e.g. [96,97]), including human [87,98] and more recently non-human [90,99] primates. This fibre type has a higher contraction velocity than MHC-1 but it is similar to MHC-1 in having high endurance and a low energy cost of activation [100,101]. Animals that execute tens of thousands of chews a day at low force amplitudes, such as marsupial grazers [96], have chewing muscles that homogeneously express MHC α-cardiac. This fibre phenotype is believed to confer a benefit to animals that chew with exceptionally high frequency but do not ruminate, because the higher rate of cross-bridge cycling compared with MHC-1 fibres [101] facilitates the rapid and efficient breakdown of food into fine particles for fermentation. MHC-M is a phylogenetically ancient myosin specific to the masticatory muscles [102] and has been functionally linked to rapid, powerful muscle and bite forces for prey capture [85].

Most single-fibre studies that have measured isometric tension in mammalian skeletal muscles have reported on MHC-1 and MHC-2 fibres in limb muscles (e.g. [64–67,103]). By contrast, isometric tension measured from single fibres that express MHC α-cardiac and MHC-M are far more limited, having been reported for the jaw muscles of cow [65] and dog and cat [64,67]. MHC α-cardiac tension in cow masseter has been reported at approximately 5 N cm^−2^ [66], lower than specific tension estimates for MHC-1 fibres. Fibres expressing MHC-M developed significantly higher isometric tension (38 N cm^−2^) when compared with MHC-1, MHC α-cardiac and MHC-2 fibres [64]. Isometric tension reported for hybrid fibres indicates that their contractile forces are intermediate relative to the combination of pure myosin isoforms expressed within that fibre [66,71]. Their intermediate tension is consistent with their other physiological characteristics, such as their contractile velocity and fatigue resistance [65,80,91,104–106].

## 2. Material and methods

### 2.1. Sample

We estimated PCSAs and determined the four major MHC isoforms present in the superficial masseter and temporalis muscles of five anthropoid primates: *Sapajus apella* (*N* = 3), *Cercocebus atys* (*N* = 4), *Macaca fascicularis* (*N* = 3), *Gorilla gorilla* (*N* = 1) and *Pan troglodytes* (*N* = 2). All tissues were from captive individuals with no evidence of craniodental or temporomandibular joint pathology or muscle atrophy. All had been fresh frozen for varying amounts of time, then thawed and fixed in 10% buffered formalin and stored in 10% buffered formalin until use. Specimens were obtained from regional primate centres or zoological institutions and no animals were sacrificed for the purposes of this study (see electronic supplementary material).

### 2.2. Measurements

#### 2.2.1. Physiological cross-sectional area

**Figure 2.**
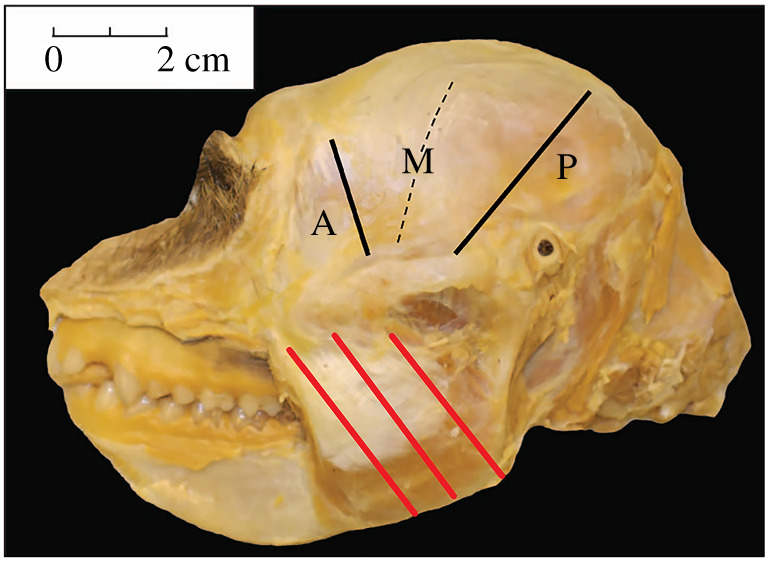
Photograph of a female *M. fascicularis* head depicting the temporalis and masseter muscles exposed *in situ*. Black lines depict the sectioning of the temporalis into anterior (A), middle (M) and posterior (P) regions. Red lines depict sections of the masseter along the length of the muscle into muscle segments. Modified from Terhune *et al*. [53].

The fibre architecture methods used in this study have been described in detail elsewhere (e.g. [30,50,51,53,55,107]). Briefly, the masseter and temporalis muscles were dissected en masse from the cranium. The deep and superficial portions of the masseter were separated and the superficial masseter and temporalis weighed to the nearest 0.01 or 0.1 g, depending on muscle size. The superficial masseter muscles were sectioned from superficial to deep along their lengths into multiple segments while the temporalis muscles were sectioned into anterior, middle and posterior segments (figure 2), using stress lines visible on the epimysium as guides (e.g. [108]). For each muscle segment, fibre length (Lf) from adjacent fibres was measured from multiple sampling sites from the superficial and intermediate compartments of the masseter (but excluding the deep masseter) and from the superficial and deep temporalis. Only intact fibres running from tendon attachment to tendon attachment were included (i.e. no cut fibres were measured). Pinnation angle (*θ*) was estimated for each fibre following Anapol & Barry [107]. We included only specimens whose jaws were fixed in comparable postures (i.e. at occlusion). Thus, for the purposes of this study (following Taylor & Vinyard [50]), we did not normalize fibre length by a standard sarcomere length.^1^

Using our estimates of muscle mass, Lf and pinnation angle, we calculated PCSA using the following equation [110]:

$$\mathrm{PCSA}\;({\mathrm{cm}}^{2})=\frac{\mathrm{muscle}\;\;\mathrm{mass}\;(g)\times \cos\theta }{\mathrm{Lf}\;(\mathrm{cm})\times \;1.0564\;(g\;{\mathrm{cm}}^{-3})},$$

where 1.0564 g cm^−3^ is an estimate of the specific density of skeletal muscle [111].

#### 2.2.2. Immunohistochemistry

We excised small blocks of muscle tissue from the anterior superficial masseter (ASM), superficial anterior temporalis (SAT) and deep anterior temporalis (DAT). The muscle blocks (approx. 8–10 mm long × approx. 8–10 mm wide × approx. 4 mm deep) were cut perpendicular to the muscle's long axis and immersed in 70% ethanol solution for 48–72 h before paraffin embedding to aid in the reconstitution of the fibres. Immunohistochemistry (IHC) sample preparation and staining were subsequently carried out in the Immunohistology Laboratory, Department of Pathology, Duke University School of Medicine, Durham, NC, USA. Tissue preservation and formalin fixation can reduce the availability of reactive sites, potentially resulting in variable and diminished staining intensity [112,113]. We thus used an IHC protocol specifically developed and tested by the Department of Pathology to improve the staining of formalin-fixed tissue [55,99,114].

Briefly, each paraffin-embedded muscle block was sectioned at 5 µm using a cryostat. Serial sections were mounted on glass microscope slides to undergo final IHC procedures. Each section was pre-treated with 1% bovine serum albumin (BSA; Sigma) and then dissolved in Tris-buffered saline, 0.1% with Tween 20 (TBST) for 20 min. Four serial sections per muscle region were stained against MHC-1 (reacting with skeletal-slow myosin; 1 : 400 dilution; NOQ7.5.4D; Sigma), MHC α-cardiac (reacting with α-cardiac-slow myosin; 1 : 400 dilution; MYH6; Sigma), MHC-2 (reacting with skeletal-fast myosin; 1 : 400 dilution; MY-32, no. 4276; Sigma; we did not differentiate among the various MHC-2 isoforms) and MHC-M (reacting with masticatory myosin; 1 : 200 dilution; 2F4; Developmental Studies Hybridoma Bank, University of Iowa) antibodies.

Photographs were taken of the resulting stained histological sections using a Nikon DS-Fi3 high-definition camera attached to a Nikon 50i microscope and NIS Elements software v. 4.5 (Nikon Instruments Inc., Melville, NY). Photographs were saved as digital images at 4× magnification and stitched together in Microsoft PowerPoint to create a complete image of each stained muscle section. Regions of interest (ROIs) were selected for counting and scoring staining intensities and for measuring cross-sectional areas (CSAs) of selected cells, ensuring that the ROIs were representative of the range of variation in staining across the muscle section.

Each ROI was photographed at 10× and imported into ImageJ v. 1.52 [115]. Using the ‘Multi-point’ tool in ImageJ, a minimum of 300 fibres was counted per muscle region for each specimen and scored for staining intensity as strong, intermediate, weak or unstained [86,90,99]. Serial sections for the same muscle region enabled us to determine if a cell stained against only one of the four antibodies (pure fibre) or two or more antibodies (hybrid fibre). Based on the antibodies used in this study, three different hybrid types were possible: (i) slow hybrids, expressing more than one slow myosin (MHC-1 and MHC α-cardiac) [116,117]; (ii) fast hybrids, expressing more than one fast myosin (MHC-2 and MHC-M) [118]; and (iii) intermediate hybrids, expressing combinations of both slow and fast myosins.

CSAs (µm^2^) of a subset of approximately 50 cells [114] per muscle region were measured using the ‘Polygon’ and ‘Freehand selection’ tools in ImageJ. Selected cells were representative of the staining patterns for each of the four antibodies for each muscle region. We used these cell CSAs to calculate the %CSA representing each fibre type expressed in our sampled muscle section.

#### 2.2.3. Muscle force estimation

To estimate maximum muscle force (MF %CSA), we used a modified version of the conventional formula where a muscle PCSA is multiplied by a muscle-specific tension [57]. Here, we multiplied the %CSA of each fibre type within a muscle by the muscle's PCSA (cm^2^), yielding a fibre-type specific estimate of PCSA (cm^2^). For example, the superficial masseter PCSA of *C. atys* ABT8 was estimated as 6.73 cm^2^ (electronic supplementary material, table S1). Of that 6.73 cm^2^, 57% %CSA was represented by the MHC-1 + MHC α-cardiac + MHC-2 hybrid fibre type (slow + 2), 20% %CSA was represented by MHC α-cardiac + MHC-2 + MHC-M hybrid fibre type (fast + α-cardiac) and 23% %CSA was represented by the MHC-1 + MHC α-cardiac hybrid fibre type (pure–slow hybrid). Each of these fibre-type specific CSAs was then multiplied by empirically derived specific tension estimates for each fibre type (as reported in table 2) and these were summed across the muscle to estimate MF %CSA (see electronic supplementary material, S1 methods and table S1). Based on the intermediate contractile properties of hybrid fibres, we averaged the known isometric tensions of all myosin isoforms expressed within a hybrid fibre to determine specific tension for that hybrid fibre type [99]:

$$\;\begin{array}{rl} & \mathrm{MF}\;\text{\%}\mathrm{CSA}=\;[(\text{\%}\mathrm{CSA}\;\mathrm{MHC}-1\times \mathrm{PCSA})\times 22.5\;N\;{\mathrm{cm}}^{-2}]\\ & +[(\text{\%}\mathrm{CSA}\;\mathrm{MHCa}-\mathrm{cardiac}\times \mathrm{PCSA})\times 5\;N\;{\mathrm{cm}}^{-2}]\\ \; & +[(\text{\%}\mathrm{CSA}\;\mathrm{MHC}-2\times \mathrm{PCSA})\times 26.3\;N\;{\mathrm{cm}}^{-2}]\\ & +\;[(\text{\%}\mathrm{CSA}\;\mathrm{MHC}-M\;\times \;\mathrm{PCSA})\times 38\;N\;{\mathrm{cm}}^{-2}]\\ & +[\left(\text{\%}\mathrm{CSA}\;\mathrm{hybrid}\times \mathrm{PCSA}\right)\times \mathrm{hybrid}\;\mathrm{isometric}\;\mathrm{tension}\;N\;{\mathrm{cm}}^{-2}]. \end{array}$$

**Table 2.** Isometric tension values (*P*_o_) used in this study.

	MHC-1	MHC α-cardiac	MHC-2	MHC-M
isometric tension (N cm^−2^)^a^	22.5	5	26.3	38

^a^All isometric tension values reported here are from single-fibre studies. *P*_o_ for MHC-1 (*N* = 8 fibres) and MCH-2 are from carnivore trunk and limb muscle [64]. The current study did not differentiate among the MHC-2 isoforms; thus, we used an average *P*_o_ of MHC-2A and MHC-2X (*N* = 32 fibres). MHC α-cardiac values are from cow (*Bos taurus*) masseter (*N* = 25 fibres) [66]. *P*_o_ for MHC-M (*N* = 23 fibres) is from carnivore masseter and temporalis muscles [64].

Studies that employ estimates of the contractile properties of muscle (e.g. *P*_o_, *V*_o_) generally report and use the mean values (for example, see O'Neill *et al*. [65], and electronic supplementary material therein). We follow this approach here. While variation in tension estimates (table 1) could impact the reported means that we used in our muscle force estimates, we believe this is no different from employing mean values of fibre length and pinnation angle that are used to estimate muscle PCSAs.

To determine the impact of proportionally adjusting muscle force estimates by their fibre types and associated specific tensions, we compared our MF %CSAs with muscle forces estimated using specific tension values for a homogeneously slow (PCSA × 22.5 N cm^−2^) and homogeneously fast muscle (PCSA × 26.3 N cm^−2^). We used the specific tension value for MHC-2 (table 2) to be consistent with most studies that have applied specific tension estimates from limb muscles. Employing these two specific tension estimates thus allows us to bracket the range of muscle force estimates typically used to estimate jaw-muscle (and bite) forces.

## 3. Results

**Figure 3.**
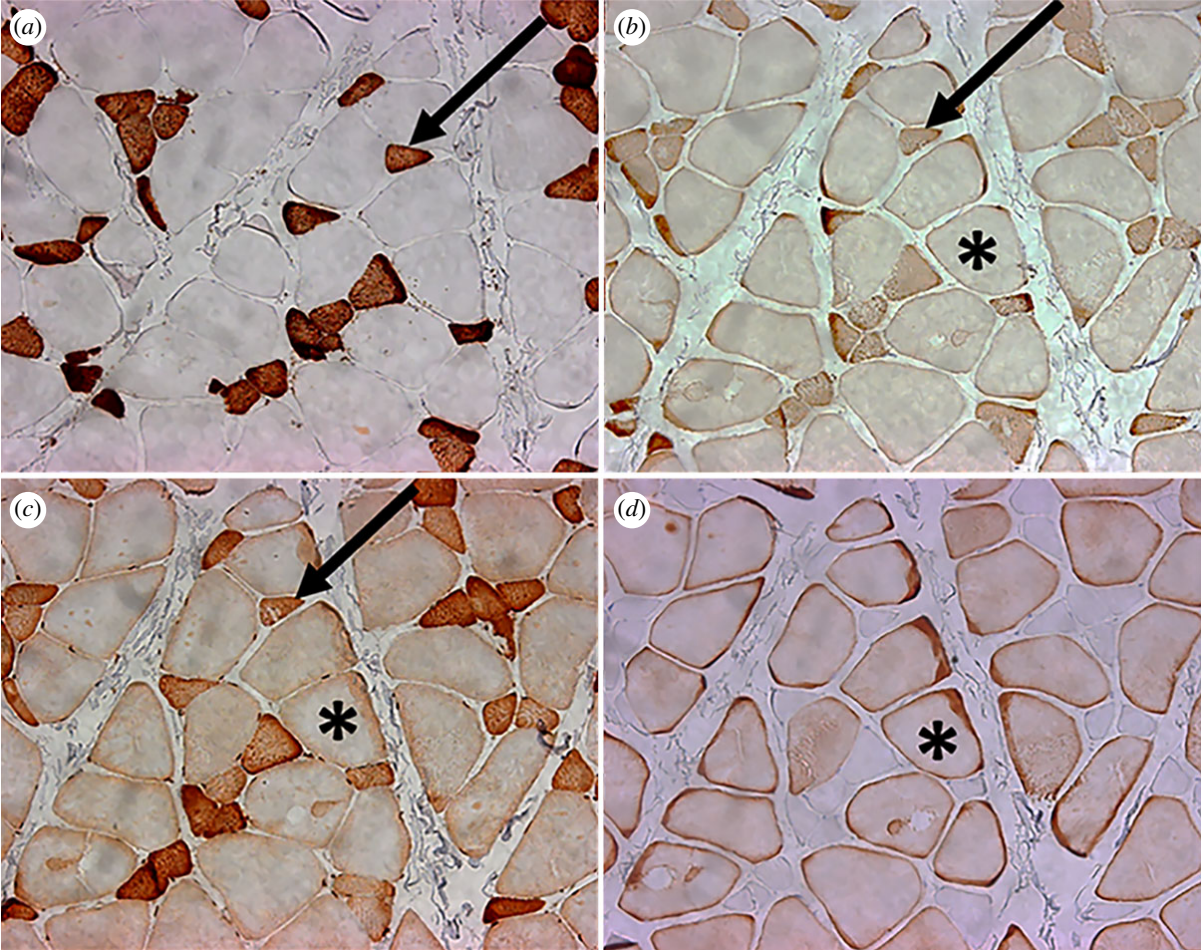
20× images of the same muscle fascicle in the anterior superficial masseter of *M. fascicularis*. (*a*) NOQ7.5.4D (MHC-1); (*b*) MYH6 (MHC α-cardiac); (*c*) MY32 (MHC-2); (*d*) (MHC-M). Note the abundance of hybrid fibres. Arrows point to the same cell co-staining with intermediate or dark intensity for MHC-1, MHC α-cardiac and MHC-2 (the slow + 2 hybrid). Asterisks indicate the same cell co-staining with light or intermediate intensity for MHC α-cardiac, MHC-2 and MHC-M (the fast + α-cardiac hybrid). Note the counter-staining between (*a*) cells that express MHC-1 and (*d*) those that express MHC-M.

**Figure 4.**
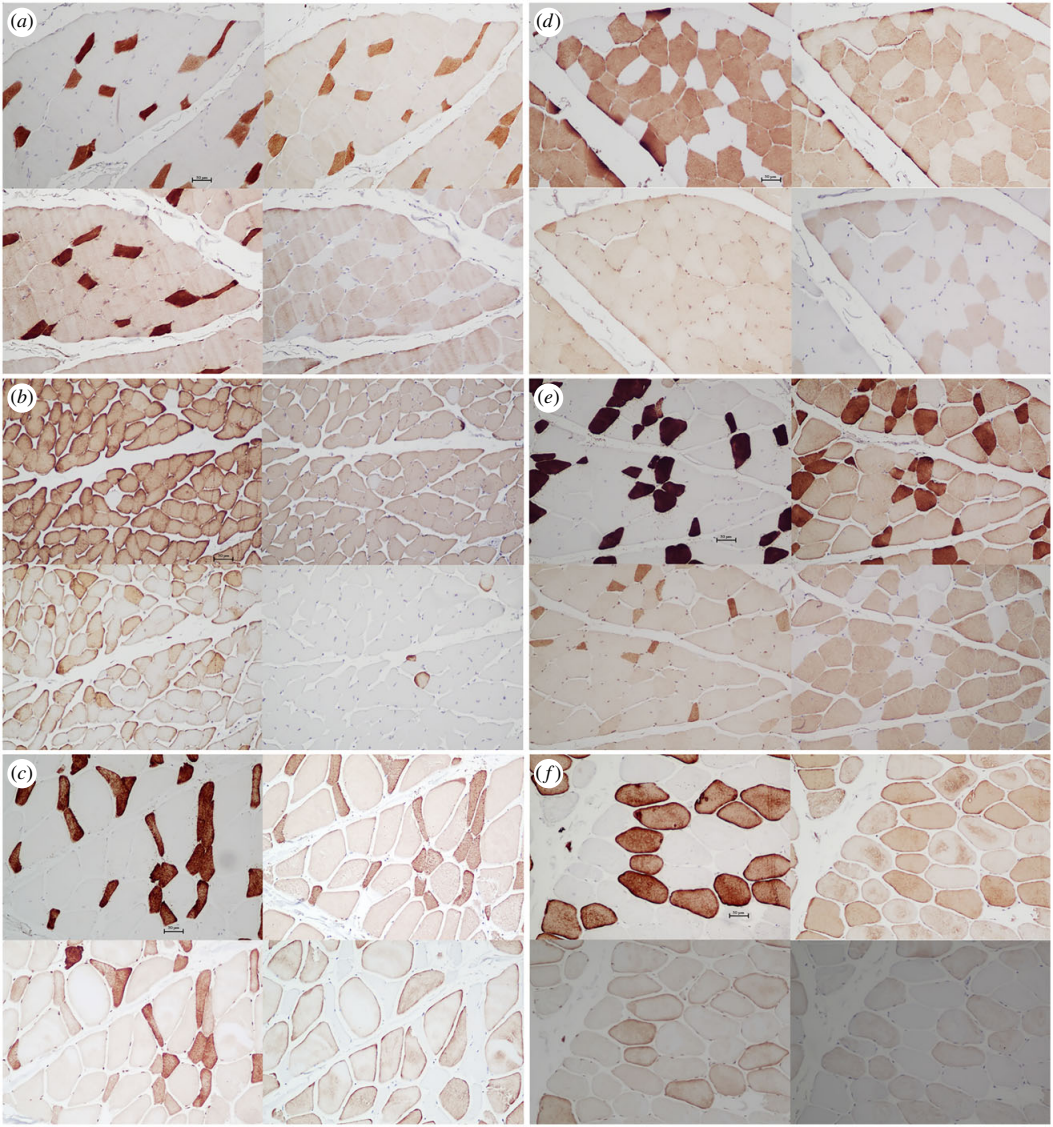
Representative serial sections for left column: (*a*) *S. apella* SAT; (*b*) *C. atys* ASM; (*c*) *M. fascicularis* DAT; right column: (*d*) *G. gorilla* ASM; (*e*) *G. gorilla* SAT; and (*f*) *P. troglodytes* ASM, stained for NOQ7.5.4D (MHC-1; upper left square), MYH6 (MHC α-cardiac; upper right square), MY-32 (MHC-2; lower left square) and 2F4 (MHC-M; lower right square). Note the ubiquitous expression of fibres co-expressing two or more MHC isoforms, the abundance of α-cardiac fibres and the consistent counterstaining between NOQ7.5.4 and 2F4. For *G. gorilla*, note the intermuscular differences in distribution and staining intensities between the ASM (*d*) and SAT (*e*; see also Taylor & Holmes [99]). All figures were taken at 10×. Scale bar, 50 µm.

Hybrid fibres were ubiquitous throughout the sampled species. Very few fibres expressed pure–slow or pure–fast phenotypes (figure 3), the single exception being the SAT in one *S. apella* (*S*. *apella* 31) that expressed 24% %CSA pure α-cardiac. The two most common hybrid combinations observed were: MHC-1 + MHC α-cardiac + MHC-2 and MHC α-cardiac + MHC-2 + MHC-M (table 3). Following Taylor & Holmes [99], we refer to these hybrid fibre phenotypes as the slow + 2 and fast + α-cardiac, respectively. The distinction between the slow + 2 and fast + α-cardiac hybrids is based on the nearly invariant counterstaining between MHC-1 and MHC-M. Other fibre types observed included MHC α-cardiac + MHC-2, pure–slow hybrid (MHC-1 + MHC α-cardiac), pure–fast hybrid (MHC-2 + MHC-M), MHC α-cardiac + MHC-M and pure MHC-2; a small percentage of fibres (less than or equal to 5%) co-stained for all four antibodies (table 3). Representative figures of the MHC expression patterns for each species are shown in figure 4.

**Table 3.** %CSA of each fibre type observed in each specimen. slow + 2 and fast + α-cardiac were the most common hybrid fibre types observed. slow + 2 = MHC-1 + MHC α-cardiac + MHC-2; fast + α-cardiac = MHC α-cardiac + MHC-2 + MHC-M; slow-hybrid = MHC-1 + MHC α-cardiac; fast-hybrid = MHC-2 + MHC-M; all = MHC-1 + MHC α-cardiac + MHC-2 + MHC-M.

species	slow + 2 (%)	fast + α-cardiac (%)	MHC α-cardiac + MHC-2 (%)	pure–slow hybrid (%)	pure–fast hybrid (%)	MHC α-cardiac + MHC-M (%)	pure MHC α-cardiac (%)	pure MHC-2 (%)	all (%)
superficial masseter									
*S. apella* 30	23	77	—	—	—	—	—	—	—
*S. apella* 31	23	62	16	—	—	—	—	—	—
*S. apella* 32	16	84	—	—	—	—	—	—	—
*C. atys* (ABT4)	98	1	—	—	—	—	—	1	—
*C. atys* (ABT5)	92	—	—	—	—	—	—	8	—
*C. atys* (ABT6)	32	67	—	—	—	—	—	—	—
*C. atys* (ABT8)	57	20	—	23	—	—	—	—	—
*M. fascicularis* (ABTWLH3)	63	3	34	—	—	—	—	—	—
*M. fascicularis* (CJV222)	39	61	—	—	—	—	—	—	—
*M. fascicularis* (CJV221)	16	84	—	—	—	—	—	—	—
*G. gorilla* (NCZ488)	94	6	—	—	—	—	—	—	—
*P. troglodytes* (CJV190)	19	76	—	—	—	—	—	—	5
*P. troglodytes* (CJV189)	23	75	2	—	—	—	—	—	—
anterior temporalis									
*S. apella* 30	18	82	—	—	—	—	—	—	—
*S. apella* 31	27	—	—	5	—	44	24	—	—
*S. apella* 32	31	66	1	1	—	—	—	—	—
*C. atys* (ABT 4)	56	—	—	—	44	—	—	—	—
*C. atys* (ABT 5)	87	—	—	—	13	—	—	—	—
*C. atys* (ABT 6)	61	39	—	—	—	—	—	—	—
*C. atys* (ABT 8)	56	44	—	—	—	—	—	—	—
*M. fascicularis* (ABTWLH3)	47	—	—	53	—	—	—	—	—
*M. fascicularis* (CJV222)	32	68	—	—	—	—	—	—	1
*M. fascicularis* (CJV221)	51	45	—	—	—	—	—	—	3
*G. gorilla* (NCZ 488)	73	27	—	—	—	—	—	—	—
*P. troglodytes* (CJV190)	16	81	—	—	—	—	3	—	—
*P. troglodytes* (CJV189)	20	60	20	—	—	—	—	—	—

Table 4 compares our estimated maximum muscle forces (MF %CSA) with muscle forces estimated assuming a homogeneously slow (MF slow = 100% MHC-1) and a homogeneously fast (MF fast = 100% MHC-2) fibre type. The percentage difference between our estimates and homogeneously slow or fast muscles ranged from 4.4% to 44.1% for the masseter and from 7.3% to 36.1% for the temporalis. Whether treated as a homogeneously slow or homogeneously fast muscle, maximum muscle forces were always greater than those estimated for our MF %CSA (the difference between MF slow and MF %CSA was negligible for *S. apella*).

**Table 4.** Average maximum muscle force estimates (N cm^−2^) for the superficial masseter and temporalis muscles assuming a homogeneously slow fibre expression (MF slow), incorporating %CSA of all fibre types expressed (MF %CSA), and assuming a homogeneously fast fibre expression (MF fast).^a–c.^

species	superficial masseter					temporalis				
	MF slow	MF %CSA	MF fast	%Dif MF %CSA versus MF slow	%Dif MF %CSA versus MF fast	MF slow	MF %CSA	MF fast	%Dif MF %CSA versus MF slow	%Dif MF %CSA versus MF fast
*S. apella*	220.9	211.5	258.2	4.4	22.1	391.6	356.2	457.8	9.9	28.5
s.d.	(39.7)	(30.8)	(46.4)			(146.0)	(166.9)	(170.6)		
*C. atys*	149.0	126.9	174.2	17.4	37.3	359.9	335.5	420.7	7.3	25.4
s.d.	(31.6)	(36.9)	(36.9)			(106.7)	(95.3)	(124.7)		
*M. fascicularis*	90.98	83.12	106.34	9.5	27.9	271.9	238.0	317.8	14.2	33.5
s.d.	(28.6)	(34.4)	(33.4)			(64.0)	(87.4)	(74.8)		
*G. gorilla*	777.7	630.7	909.1	23.3	44.1	1272.4	1092.8	1487.3	16.4	36.1
s.d.	—	—	—			—	—	—		
*P. troglodytes*	813.3	792.2	950.6	2.7	20.0	1132.8	1065.6	1324.1	6.3	24.3
s.d.	—	—	—			—	—	—		

^a^MF slow: muscle force estimates based on 100% MHC-1 with *P*_o_ = 22.5 N cm^−2^; MF fast: muscle force estimates based on 100% MHC-2 with *P*_o_ = 26.3 N cm^−2^; %Dif: % difference; s.d., standard deviation.

^b^Values are averaged by muscle for each species.

^c^% difference calculated by subtracting MF slow or MF fast from MF %CSA, taking the absolute value (ABS) of that difference, dividing by MF %CSA and multiplying by 100, e.g. ABS [(MF %CSA − MF slow)/(MF %CSA)] * 100.

## 4. Discussion and conclusion

Anthropological studies that have estimated maximum muscle and bite forces in extant human and non-human primates have generally applied either a slow-type (e.g. 22.5 N cm^−2^) [51] or fast-type (e.g. [16,119,120]) specific tension value uniformly across the chewing muscles; faster tension values have also been applied to studies that have modelled bite forces in extinct primates and non-primate mammals (e.g. [21,54,121]). Importantly, tension values applied to the jaw adductors derive exclusively from work on non-primate mammalian limb muscles (e.g. [57,59,83]).

We show that average maximum jaw-muscle force estimates that account for fibre-type proportions and their fibre-specific tensions (MF %CSA) are smaller by as much as 30% than those assigned a single specific tension across the entire muscle (table 4). As might be expected, these differences in jaw-muscle force estimates translate into differences in bite force (see electronic supplementary material). At first glance, it may seem counterintuitive that our fibre-type specific tension estimates of muscle force are lower than those obtained when assigning specific tensions that are either homogeneously slow or fast. This pattern is explained by the fact that MHC α-cardiac is abundantly expressed across both the superficial masseter and temporalis in all our sample species, consistent with previous studies of non-primate mammals (e.g. [122]), as well as human [98] and non-human primates [90,99]. The reported *P*_o_ of approximately 5 N cm^−2^ for MHC α-cardiac [66] is lower than the *P*_o_ reported for MHC-1 (e.g. [64–67] and references therein). Incorporating the high proportion of fibres expressing MHC α-cardiac in our samples, with its lower *P*_o_, thus lowers the maximum muscle force estimates compared with those obtained when using even a slow-type specific tension.

We used mean *P*_o_ values estimated from single fibres in our muscle force estimates, following previous muscle performance studies (e.g. [65]). As with all estimates of muscle kinetic and contractile properties, as well as input parameters of muscle PCSA (e.g. [47]), the mean values used in this study exhibit a range (e.g. [64,66]). We were unable to bracket the range of specific tension for each of the four MHC isoforms used in the current study as these were not explicitly reported by Toniolo *et al*. [64,66]. These authors do report significant differences in mean *P*_o_ between MHC-1 and MHC-M, and between MHC 2A and MHC-M [64]. The *P*_o_ for MHC α-cardiac is roughly 25% of that of MHC-1 [64,66], suggesting this value likely differs significantly from MHC-1, MHC-2A, MHC-2X and MHC-M. We are thus reasonably confident that the variation around the mean *P*_o_ values within each MHC isoform is substantially lower than the variation between MHC isoforms. When *P*_o_ is estimated from single fibres from the same muscle using the same experimental protocol, as is the case in the work reported by Toniolo *et al*. [64,67], the mean can be expected to represent the best estimate. Future work to explore variation in contractile properties and how it effects muscle force estimates could be better addressed by resampling techniques where the ranges of specific tension could be explicitly incorporated in the model.

Fibre-type adjusted jaw-muscle PCSAs (like PCSAs unadjusted for fibre type) represent static estimates of the relative force contribution of each chewing muscle to total jaw-adductor muscle force. Our focus in this study is to further refine static muscle force estimates by incorporating an additional and important parameter—the *P*_o_ of the various fibre types that make up a given muscle and their proportions—and to evaluate the impact of accounting for various fibre types on these muscle force estimates. These parameters do not account for the fact that the jaw adductors fire both synchronously and asynchronously during chewing [123,124]. However, when combined with estimates of jaw-muscle mechanical advantage, they can be used to estimate maximum isometric bite force anywhere along the tooth row using static models (e.g. [10–12]) and have been fruitfully applied to biomechanical and evolutionary questions of feeding behaviour and diet in a wide range of extant primates (e.g. [16,17,33]) and non-primate mammals (e.g. [36]) and in palaeobiological contexts (e.g. [125]).

Similar to other studies of mammalian jaw-muscle fibre types (e.g. [86,88,90,98,99]), hybrid fibres were ubiquitous in our primate sample and fibres that expressed MHC α-cardiac generally co-expressed MHC-1 (e.g. [122]) as well as MHC-2 (figure 3; see also [90,99]). As such, hybrid fibres were proportionally averaged across all the MHC isoforms expressed. It is also important to point out that, to date, mammalian single-fibre specific tension values reported for MHC-1 and MHC-2 are known only from limb and trunk muscles; to our knowledge, these have not been reported from the chewing muscles of any mammal. We employed a *P*_o_ for MHC-1 of 22.5 N cm^−2^ [64], which is higher than those reported for humans (e.g. [65] and references therein) and at least some other mammals (e.g. [64,66,67,74]). Had we employed a specific tension for MHC-1 closer to those reported for humans (many report *P*_o_ approx. 10–18 N cm^−2^; [65] and references therein), our MF %CSAs would have been lower still.

The variation reported across specific tension estimates is likely to be due to a number of factors. While there is no clear correlation between specific tension and body mass [65,73,74], some of these differences can be attributed to variation across muscles and species (table 1). Different methods used to measure maximum isometric muscle force (*P*_o_) are also likely to account for some of this variation. For example, experimental measurements of maximum isometric muscle force (*P*_o_) are temperature dependent [126] as well as dependent on tissue preparation, e.g. whether estimated from skinned single muscle fibres (e.g. [127]) versus from stimulated intact whole muscle (e.g. [59]). Measuring isometric tension from single fibres of primate chewing muscles has proven challenging (P. Reiser 2019, personal communication). Given this, valuable next steps would include measuring isometric tension of single fibres expressing MHC-1 and MHC-2 from a variety of mammalian chewing muscles to determine if their contractile properties are similar to those expressed in limb muscles. Likewise, estimating *P*_o_ from mammalian chewing muscles that express MHC α-cardiac and hybrid combinations would further contribute to refining estimates of maximum muscle forces.

Our results for a select subset of anthropoid primates suggest that fibre types have the potential to strongly impact muscle (and bite) force estimates. Accounting for the intra- and intermuscular heterogeneity of primate jaw-muscle fibre phenotypes is thus likely to improve muscle and bite forces estimates in extant taxa. While fibre types cannot be directly estimated in palaeontological specimens, skeletal estimates of bite force have been modelled in extinct hominins by applying correction factors based in part on architectural estimates of jaw-muscle PCSAs derived from extant primates (e.g. Eng *et al*. [16]). Adjusting these PCSAs by fibre type in extant taxa may thus improve bite force estimates in palaeobiological contexts, particularly if the bite force estimates in extant taxa can be validated by *in vivo* bite force studies.

## Endnote

1 Skeletal muscle fibre length is influenced by joint posture and normalizing fibre length by a standard sarcomere length has been empirically shown to eliminate this error [89,109]. Gape-dependent variation in fibre lengths of the chewing muscles is effectively eliminated when normalizing measured fibre lengths by a sarcomere length of 2.41 μm, such that normalized fibre lengths are not significantly different (*p* < 0.05) from fibre lengths measured at occlusion [109]. In the absence of gape-dependent variation in our sample, we thus report PCSAs based on non-normalized fibre lengths.

## Data accessibility

Additional data are provided in the electronic supplementary material [128].

## References

[1] Hylander WL, Bays R. 1979 An in vivo strain-gauge analysis of the squamosal-dentary joint reaction force during mastication and incisal biting in *Macaca mulatta* and *Macaca fascicularis*. Arch. Oral Biol. **24**, 689-697. (10.1016/0003-9969(79)90119-5) 120172

[2] Hylander WL. 1979 The functional significance of primate mandibular form. J. Morphol. **160**, 223-239. (10.1002/jmor.1051600208) 458862

[3] Hylander WL. 1979 Mandibular function in *Galago crassicaudatus* and *Macaca fascicularis*: an in vivo approach to stress analysis of the mandible. J. Morphol. **159**, 253-296. (10.1002/jmor.1051590208) 105147

[4] Cole III TM. 1992 Postnatal heterochrony of the masticatory apparatus in *Cebus apella* and *Cebus albifrons*. J. Hum. Evol. **23**, 253-282. (10.1016/S0047-2484(05)80003-X)

[5] Daegling DJ. 1989 Biomechanics of cross-sectional size and shape in the hominoid mandibular corpus. Am. J. Phys. Anthropol. **80**, 91-106. (10.1002/ajpa.1330800111) 2508480

[6] Daegling DJ. 1992 Mandibular morphology and diet in the genus *Cebus*. Int. J. Primatol. **13**, 545-570. (10.1007/BF02547832)

[7] Hylander WL. 1988 Implications of in vivo experiments for interpreting the functional significance of ‘robust’ australopithecine jaws. In Evolutionary history of the ‘robust’ australopithecines (ed. F Grine), pp. 55-83. New York, NY: Taylor and Francis.

[8] Ravosa MJ. 2000 Size and scaling in the mandible of living and extinct apes. Folia Primatol. **71**, 305-322. (10.1159/000021754) 11093035

[9] Vinyard CJ, Ravosa MJ. 1998 Ontogeny, function, and scaling of the mandibular symphysis in papionin primates. J. Morphol. **235**, 157-175. (10.1002/(SICI)1097-4687(199802)235:2<157::AID-JMOR5>3.0.CO;2-6) 9438974

[10] Greaves W. 1978 The jaw lever system in ungulates: a new model. J. Zool. **184**, 271-285. (10.1111/j.1469-7998.1978.tb03282.x)

[11] Hylander WL. 1975 The human mandible: lever or link? Am. J. Phys. Anthropol. **43**, 227-242. (10.1002/ajpa.1330430209) 1101706

[12] Spence MA, Demes B. 1993 Biomechanical analysis of masticatory system configuration in Neandertals and Inuits. Am. J. Phys. Anthropol. **91**, 1-20. (10.1002/ajpa.1330910102) 8512051

[13] Dechow PC, Carlson DS. 1990 Occlusal force and craniofacial biomechanics during growth in rhesus monkeys. Am. J. Phys. Anthropol. **83**, 219-237. (10.1002/ajpa.1330830211) 2248381

[14] Demes B, Creel N. 1988 Bite force, diet, and cranial morphology of fossil hominids. J. Hum. Evol. **17**, 657-670. (10.1016/0047-2484(88)90023-1)

[15] Deutsch AR, Dickinson E, Leonard KC, Pastor F, Muchlinski MN, Hartstone-Rose A. 2020 Scaling of anatomically derived maximal bite force in primates. Anat. Rec. **303**, 2026-2035. (10.1002/ar.24284) 31587507

[16] Eng CM, Lieberman DE, Zink KD, Peters MA. 2013 Bite force and occlusal stress production in hominin evolution. Am. J. Phys. Anthropol. **151**, 544-557. (10.1002/ajpa.22296) 23754526

[17] Norconk MA, Wright BW, Conklin-Brittain NL, Vinyard CJ. 2009 Mechanical and nutritional properties of food as factors in platyrrhine dietary adaptations. In South American primates (eds PA Garber, A Estrada, JC Bicca-Marques, EW Heymann, KB Strie), pp. 279-319. Berlin, Germany: Springer.

[18] Wright BW. 2005 Craniodental biomechanics and dietary toughness in the genus *Cebus*. J. Hum. Evol. **48**, 473-492. (10.1016/j.jhevol.2005.01.006) 15857651

[19] Panagiotopoulou O, Iriarte-Diaz J, Wilshin S, Dechow PC, Taylor AB, Abraha HM, Aljunid SF, Ross CF. 2017 In vivo bone strain and finite element modeling of a rhesus macaque mandible during mastication. Zoology **124**, 13-29. (10.1016/j.zool.2017.08.010) 29037463 PMC5792078

[20] Strait DS, Richmond BG, Spencer A, Ross CF, Dechow PC, Wood BA. 2007 Masticatory biomechanics and its relevance to early hominid phylogeny: an examination of palatal thickness using finite-element analysis. J. Hum. Evol. **52**, 585-599. (10.1016/j.jhevol.2006.11.019) 17386938

[21] Strait DS et al. 2009 The feeding biomechanics and dietary ecology of *Australopithecus africanus*. Proc. Natl Acad. Sci. USA **106**, 2124-2129. (10.1073/pnas.0808730106) 19188607 PMC2650119

[22] Wroe S, Moreno K, Clausen P, McHenry C, Curnoe D. 2007 High-resolution three-dimensional computer simulation of hominid cranial mechanics. Anat. Rec. **290**, 1248-1255. (10.1002/ar.20594) 17847056

[23] Gröning F, Liu J, Fagan MJ, O'Higgins P. 2011 Why do humans have chins? Testing the mechanical significance of modern human symphyseal morphology with finite element analysis. Am. J. Phys. Anthropol. **144**, 593-606. (10.1002/ajpa.21447) 21404235

[24] Ledogar JA, Luk TH, Perry JM, Neaux D, Wroe S. 2018 Biting mechanics and niche separation in a specialized clade of primate seed predators. PLoS ONE **13**, e0190689. (10.1371/journal.pone.0190689) 29324822 PMC5764286

[25] Panagiotopoulou O, Iriarte-Diaz J, Abraha HM, Taylor AB, Wilshin S, Dechow PC, Ross CF. 2020 Biomechanics of the mandible of *Macaca mulatta* during the power stroke of mastication: loading, deformation, and strain regimes and the impact of food type. J. Hum. Evol. **147**, 102865. (10.1016/j.jhevol.2020.102865) 32905895 PMC7541691

[26] Ross CF, Berthaume MA, Dechow PC, Iriarte-Diaz J, Porro LB, Richmond BG, Spencer M, Strait D. 2011 In vivo bone strain and finite-element modeling of the craniofacial haft in catarrhine primates. J. Anat. **218**, 112-141. (10.1111/j.1469-7580.2010.01322.x) 21105871 PMC3039785

[27] Gröning F, Fagan M, O'Higgins P. 2012 Modeling the human mandible under masticatory loads: which input variables are important? Anat. Rec. **295**, 853-863. (10.1002/ar.22455) 22467624

[28] Ross CF, Patel BA, Slice DE, Strait DS, Dechow PC, Richmond BG, Spencer MA. 2005 Modeling masticatory muscle force in finite element analysis: sensitivity analysis using principal coordinates analysis. Anat. Rec. **283**, 288-299. (10.1002/ar.a.20170) 15747351

[29] Strait DS, Wang Q, Dechow PC, Ross CF, Richmond BG, Spencer MA, Patel BA. 2005 Modeling elastic properties in finite-element analysis: how much precision is needed to produce an accurate model? Anat. Rec. **283**, 275-287. (10.1002/ar.a.20172) 15747346

[30] Anapol F, Shahnoor N, Ross CF. 2008 Scaling of reduced physiologic cross-sectional area in primate muscles of mastication. In Primate craniofacial function and biology (eds C Vinyard, R Matthew, C Wall), pp. 201-216. Berlin, Germany: Springer.

[31] Antón S. 1999 Macaque masseter muscle: internal architecture, fiber length and cross-sectional area. Int. J. Primatol. **20**, 441-462. (10.1023/A:1020509006259)

[32] Antón SC. 2000 Macaque pterygoid muscles: internal architecture, fiber length, and cross-sectional area. Int. J. Primatol. **21**, 131-156. (10.1023/A:1005431831444)

[33] Perry JM, Hartstone-Rose A, Wall CE. 2011 The jaw adductors of strepsirrhines in relation to body size, diet, and ingested food size. Anat. Rec. **294**, 712-728. (10.1002/ar.21354) 21365776

[34] Taylor AB, Vinyard CJ. 2013 The relationships among jaw-muscle fiber architecture, jaw morphology, and feeding behavior in extant apes and modern humans. Am. J. Phys. Anthropol. **151**, 120-134. (10.1002/ajpa.22260) 23553609

[35] Martin ML, Travouillon KJ, Fleming PA, Warburton NM. 2020 Review of the methods used for calculating physiological cross-sectional area (PCSA) for ecological questions. J. Morphol. **281**, 778-789. (10.1002/jmor.21139) 32374505

[36] Herrel A, De Smet A, Aguirre LF, Aerts P. 2008 Morphological and mechanical determinants of bite force in bats: do muscles matter? J. Exp. Biol. **211**, 86-91. (10.1242/jeb.012211) 18083736

[37] Mara KR, Motta PJ, Huber DR. 2010 Bite force and performance in the durophagous bonnethead shark, *Sphyrna tiburo*. J. Exp. Zool. **313**, 95-105. (10.1002/jez.576) 19844984

[38] Williams SH, Peiffer E, Ford S. 2009 Gape and bite force in the rodents *Onychomys leucogaster* and *Peromyscus maniculatus*: does jaw-muscle anatomy predict performance? J. Morphol. **270**, 1338-1347. (10.1002/jmor.10761) 19480012

[39] Erickson GM, Lappin AK, Vliet KA. 2003 The ontogeny of bite-force performance in American alligator (*Alligator mississippiensis*). J. Zool. **260**, 317-327. (10.1017/S0952836903003819)

[40] Herrel A, Spithoven L, Van Damme R, De Vree F. 1999 Sexual dimorphism of head size in *Gallotia galloti*: testing the niche divergence hypothesis by functional analyses. Funct. Ecol. **13**, 289-297. (10.1046/j.1365-2435.1999.00305.x)

[41] Verwaijen D, Van Damme R, Herrel A. 2002 Relationships between head size, bite force, prey handling efficiency and diet in two sympatric lacertid lizards. Funct. Ecol. **16**, 842-850. (10.1046/j.1365-2435.2002.00696.x)

[42] Dickinson E, Kolli S, Schwenk A, Davis CE, Hartstone-Rose A. 2020 DiceCT analysis of the extreme gouging adaptations within the masticatory apparatus of the aye-aye (*Daubentonia madagascariensis*). Anat. Rec. **303**, 282-294. (10.1002/ar.24303) 31714689

[43] Leonard KC, Boettcher ML, Dickinson E, Malhotra N, Aujard F, Herrel A, Hartstone-Rose A. 2020 The ontogeny of masticatory muscle architecture in *Microcebus murinus*. Anat. Rec. **303**, 1364-1373. (10.1002/ar.24259) 31509342

[44] Perry JM, Macneill KE, Heckler AL, Rakotoarisoa G, Hartstone-Rose A. 2014 Anatomy and adaptations of the chewing muscles in Daubentonia (Lemuriformes). Anat. Rec. **297**, 308-316. (10.1002/ar.22844) 24339191

[45] Perry JM, Wall CE. 2008 Scaling of the chewing muscles in prosimians. In Primate craniofacial function and biology (eds CJ Vinyard, M Ravosa, C Wall), pp. 217-240. Boston, MA: Springer.

[46] Perry JMG. 2008 The anatomy of mastication in extant strepsirrhines and Eocene adapines. PhD dissertation, Duke University, Durham, NC, USA.

[47] Dickinson E, Fitton LC, Kupczik K. 2018 Ontogenetic changes to muscle architectural properties within the jaw-adductor musculature of *Macaca fascicularis*. Am. J. Phys. Anthropol. **167**, 291-310. (10.1002/ajpa.23628) 30168867

[48] Dickinson E, Stark H, Kupczik K. 2018 Non-destructive determination of muscle architectural variables through the use of dice CT. Anat. Rec. **301**, 363-377. (10.1002/ar.23716) 29330959

[49] Taylor AB, Eng CM, Anapol FC, Vinyard CJ. 2009 The functional correlates of jaw-muscle fiber architecture in tree-gouging and nongouging callitrichid monkeys. Am. J. Phys. Anthropol. **139**, 353-367. (10.1002/ajpa.20991) 19140215

[50] Taylor AB, Vinyard CJ. 2004 Comparative analysis of masseter fiber architecture in tree-gouging (*Callithrix jacchus*) and nongouging (*Saguinus oedipus*) callitrichids. J. Morphol. **261**, 276-285. (10.1002/jmor.10249) 15281057

[51] Taylor AB, Vinyard CJ. 2009 Jaw-muscle fiber architecture in tufted capuchins favors generating relatively large muscle forces without compromising jaw gape. J. Hum. Evol. **57**, 710-720. (10.1016/j.jhevol.2009.06.001) 19875148 PMC3082281

[52] Taylor AB, Yuan T, Ross CF, Vinyard CJ. 2015 Jaw-muscle force and excursion scale with negative allometry in platyrrhine primates. Am. J. Phys. Anthropol. **158**, 242-256. (10.1002/ajpa.22782) 26175006

[53] Terhune CE, Hylander WL, Vinyard CJ, Taylor AB. 2015 Jaw-muscle architecture and mandibular morphology influence relative maximum jaw gapes in the sexually dimorphic *Macaca fascicularis*. J. Hum. Evol. **82**, 145-158. (10.1016/j.jhevol.2015.02.006) 25858337

[54] O'Connor CF, Franciscus RG, Holton NE. 2005 Bite force production capability and efficiency in Neandertals and modern humans. Am. J. Phys. Anthropol. **127**, 129-151. (10.1002/ajpa.20025) 15558614

[55] Taylor AB, Terhune CE, Toler M, Holmes M, Ross CF, Vinyard CJ. 2018 Jaw-muscle fiber architecture and leverage in the hard-object feeding sooty mangabey are not structured to facilitate relatively large bite forces compared to other papionins. Anat. Rec. **301**, 325-342. (10.1002/ar.23718) 29330952

[56] Perry JM. 2018 Inferring the diets of extinct giant lemurs from osteological correlates of muscle dimensions. Anat. Rec. **301**, 343-362. (10.1002/ar.23719) 29330948

[57] Powell PL, Roy RR, Kanim P, Bello MA, Edgerton VR. 1984 Predictability of skeletal muscle tension from architectural determinations in guinea pig hindlimbs. J. Appl. Physiol. **57**, 1715-1721. (10.1152/jappl.1984.57.6.1715) 6511546

[58] Roy RR, Meadows ID, Baldwin KM, Edgerton VR. 1982 Functional significance of compensatory overloaded rat fast muscle. J. Appl. Physiol. **52**, 473-478. (10.1152/jappl.1982.52.2.473) 7061301

[59] Spector SA, Gardiner PF, Zernicke RF, Roy RR, Edgerton VR. 1980 Muscle architecture and force-velocity characteristics of cat soleus and medial gastrocnemius: implications for motor control. J. Neurophysiol. **44**, 951-960. (10.1152/jn.1980.44.5.951) 7441324

[60] Wickiewicz TL, Roy RR, Powell PL, Perrine JJ, Edgerton VR. 1984 Muscle architecture and force-velocity relationships in humans. J. Appl. Physiol. **57**, 435-443. (10.1152/jappl.1984.57.2.435) 6469814

[61] Lucas S, Ruff R, Binder M. 1987 Specific tension measurements in single soleus and medial gastrocnemius muscle fibers of the cat. Exp. Neurol. **95**, 142-154. (10.1016/0014-4886(87)90013-6) 2947808

[62] Dum R, Burke R, O'Donovan M, Toop J, Hodgson J. 1982 Motor-unit organization in flexor digitorum longus muscle of the cat. J. Neurophysiol. **47**, 1108-1125. (10.1152/jn.1982.47.6.1108) 7108574

[63] Bodine S, Roy RR, Eldred E, Edgerton VR. 1987 Maximal force as a function of anatomical features of motor units in the cat tibialis anterior. J. Neurophysiol. **57**, 1730-1745. (10.1152/jn.1987.57.6.1730) 3598628

[64] Toniolo L, Cancellara P, Maccatrozzo L, Patruno M, Mascarello F, Reggiani C. 2008 Masticatory myosin unveiled: first determination of contractile parameters of muscle fibers from carnivore jaw muscles. Am. J. Physiol. Cell Physiol. **295**, C1535-C1542. (10.1152/ajpcell.00093.2008) 18842829

[65] O'Neill MC, Umberger BR, Holowka NB, Larson SG, Reiser PJ. 2017 Chimpanzee super strength and human skeletal muscle evolution. Proc. Natl Acad. Sci. USA **114**, 7343-7348. (10.1073/pnas.1619071114) 28652350 PMC5514706

[66] Toniolo L, Maccatrozzo L, Patruno M, Caliaro F, Mascarello F, Reggiani C. 2005 Expression of eight distinct MHC isoforms in bovine striated muscles: evidence for MHC-2B presence only in extraocular muscles. J. Exp. Biol. **208**, 4243-4253. (10.1242/jeb.01904) 16272247

[67] Toniolo L et al. 2007 Fiber types in canine muscles: myosin isoform expression and functional characterization. Am. J. Physiol. Cell Physiol. **292**, C1915-C1926. (10.1152/ajpcell.00601.2006) 17251320

[68] Gilliver S, Degens H, Rittweger J, Sargeant A, Jones D. 2009 Variation in the determinants of power of chemically skinned human muscle fibres. Exp. Physiol. **94**, 1070-1078. (10.1113/expphysiol.2009.048314) 19638363

[69] Larsson L, Moss R. 1993 Maximum velocity of shortening in relation to myosin isoform composition in single fibres from human skeletal muscles. J. Physiol. **472**, 595-614. (10.1113/jphysiol.1993.sp019964) 8145163 PMC1160504

[70] Paoli A, Pacelli QF, Cancellara P, Toniolo L, Moro T, Canato M, Miotti D, Reggiani C. 2013 Myosin isoforms and contractile properties of single fibers of human latissimus dorsi muscle. BioMed Res. Int. **205**, 2203-2210. (10.1155/2013/249398) PMC3736486 23971027

[71] Bottinelli R, Canepari M, Pellegrino M, Reggiani C. 1996 Force-velocity properties of human skeletal muscle fibres: myosin heavy chain isoform and temperature dependence. J. Physiol. **495**, 573-586. (10.1113/jphysiol.1996.sp021617) 8887767 PMC1160815

[72] Andruchov O, Andruchova O, Wang Y, Galler S. 2004 Kinetic properties of myosin heavy chain isoforms in mouse skeletal muscle: comparison with rat, rabbit, and human and correlation with amino acid sequence. Am. J. Physiol. Cell Physiol. **287**, C1725-C1732. (10.1152/ajpcell.00255.2004) 15306546

[73] Seow C, Ford L. 1991 Shortening velocity and power output of skinned muscle fibers from mammals having a 25,000-fold range of body mass. J. Gen. Physiol. **97**, 541-560. (10.1085/jgp.97.3.541) 2037839 PMC2216485

[74] Pellegrino M, Canepari M, Rossi R, D'Antona G, Reggiani C, Bottinelli R. 2003 Orthologous myosin isoforms and scaling of shortening velocity with body size in mouse, rat, rabbit and human muscles. J. Physiol. **546**, 677-689. (10.1113/jphysiol.2002.027375) 12562996 PMC2342590

[75] Lynch GS, Frueh BR, Williams DA. 1994 Contractile properties of single skinned fibres from the extraocular muscles, the levator and superior rectus, of the rabbit. J. Physiol. **475**, 337-346. (10.1113/jphysiol.1994.sp020074) 8021839 PMC1160383

[76] Bottinelli R, Schiaffino S, Reggiani C. 1991 Force-velocity relations and myosin heavy chain isoform compositions of skinned fibres from rat skeletal muscle. J. Physiol. **437**, 655-672. (10.1113/jphysiol.1991.sp018617) 1890654 PMC1180069

[77] Close R. 1972 Dynamic properties of mammalian skeletal muscles. Physiol. Rev. **52**, 129-197. (10.1152/physrev.1972.52.1.129) 4256989

[78] Gans C, Gaunt AS. 1991 Muscle architecture in relation to function. J. Biomech. **24**, 53-65. (10.1016/0021-9290(91)90377-Y) 1791182

[79] Bodine SC, Roy R, Meadows D, Zernicke R, Sacks R, Fournier M, Edgerton V. 1982 Architectural, histochemical, and contractile characteristics of a unique biarticular muscle: the cat semitendinosus. J. Neurophysiol. **48**, 192-201. (10.1152/jn.1982.48.1.192) 7119845

[80] Reiser PJ, Moss R, Giulian GG, Greaser ML. 1985 Shortening velocity in single fibers from adult rabbit soleus muscles is correlated with myosin heavy chain composition. J. Biol. Chem. **260**, 9077-9080. (10.1016/S0021-9258(17)39330-4) 4019463

[81] Schiaffino S, Reggiani C. 2011 Fiber types in mammalian skeletal muscles. Physiol. Rev. **91**, 1447-1531. (10.1152/physrev.00031.2010) 22013216

[82] Anapol FC, Jungers WL. 1986 Architectural and histochemical diversity within the quadriceps femoris of the brown lemur (*Lemur fulvus*). Am. J. Phys. Anthropol. **69**, 355-375. (10.1002/ajpa.1330690308) 3706515

[83] Sacks RD, Roy RR. 1982 Architecture of the hind limb muscles of cats: functional significance. J. Morphol. **173**, 185-195. (10.1002/jmor.1051730206) 7120421

[84] Hoh JF. 2002 ‘Superfast’ or masticatory myosin and the evolution of jaw-closing muscles of vertebrates. J. Exp. Biol. **205**, 2203-2210. (10.1242/jeb.205.15.2203) 12110654

[85] Rowlerson A, Mascarello F, Veggetti A, Carpenè E. 1983 The fibre-type composition of the first branchial arch muscles in Carnivora and Primates. J. Muscle Res. Cell Motil. **4**, 443-472. (10.1007/BF00711949) 6355175

[86] Wall CE, Briggs MM, Huq E, Hylander WL, Schachat F. 2013 Regional variation in IIM myosin heavy chain expression in the temporalis muscle of female and male baboons (*Papio anubis*). Arch. Oral Biol. **58**, 435-443. (10.1016/j.archoralbio.2012.09.008) 23102552 PMC3593987

[87] Korfage JA, Koolstra JH, Langenbach GE, Van Eijden TM. 2005 Fiber-type composition of the human jaw muscles—(part 2) role of hybrid fibers and factors responsible for inter-individual variation. J. Dent. Res. **84**, 784-793. (10.1177/154405910508400902) 16109985

[88] Medler S. 2019 Mixing it up: the biological significance of hybrid skeletal muscle fibers. J. Exp. Biol. **222**, 200832. (10.1242/jeb.200832) 31784473

[89] Taylor AB, Terhune CE, Vinyard CJ. 2019 The influence of masseter and temporalis sarcomere length operating ranges as determined by laser diffraction on architectural estimates of muscle force and excursion in macaques (*Macaca fascicularis* and *Macaca mulatta*). Arch. Oral Biol. **105**, 35-45. (10.1016/j.archoralbio.2019.05.015) 31254839 PMC6739116

[90] Wall CE, Holmes M, Soderblom EJ, Taylor AB. 2018 Proteomics and immunohistochemistry identify the expression of α-cardiac myosin heavy chain in the jaw-closing muscles of sooty mangabeys (order Primates). Arch. Oral Biol. **91**, 103-108. (10.1016/j.archoralbio.2018.01.019) 29703519

[91] Pette D. 2006 Skeletal muscle plasticity–history, facts and concepts. In Skeletal muscle plasticity in health and disease (eds R Bottinelli, C Reggiani), pp. 1-27. Berlin, Germany: Springer.

[92] Andreo J, Oliveira J, Navarro J, Roque D, Roque J, Buchain R. 2002 Histoenzymology and morphometry of the masticatory muscles of tufted capuchin monkey (*Cebus apella* Linnaeus, 1758). Okajimas Folia Anat. Jpn. **79**, 33-41. (10.2535/ofaj.79.33) 12199536

[93] Maxwell LC, Carlson DS, McNamara Jr JA, Faulkner JA. 1979 Histochemical characteristics of the masseter and temporalis muscles of the rhesus monkey (*Macaca mulatta*). Anat. Rec. **193**, 389-401. (10.1002/ar.1091930306) 154857

[94] Miller AJ, Farias M. 1988 Histochemical and electromyographic analysis of craniomandibular muscles in the rhesus monkey, *Macaca mulatta*. J. Oral Maxillofac. Surg. **46**, 767-776. (10.1016/0278-2391(88)90187-5) 3166047

[95] Bredman J, Wessels A, Weijs W, Korfage J, Soffers C, Moorman A. 1991 Demonstration of ‘cardiac-specific’ myosin heavy chain in masticatory muscles of human and rabbit. Histochem. J. **23**, 160-170. (10.1007/BF01046587) 1836206

[96] Hoh JF, Kim Y, Sieber LG, Zhong WW, Lucas CA. 2000 Jaw-closing muscles of kangaroos express α-cardiac myosin heavy chain. J. Muscle Res. Cell Motil. **21**, 673-680. (10.1023/A:1005676106940) 11227794

[97] Sciote J, Rowlerson A, Hopper C, Hunt N. 1994 Fibre type classification and myosin isoforms in the human masseter muscle. J. Neurol. Sci. **126**, 15-24. (10.1016/0022-510X(94)90089-2) 7836942 PMC3863992

[98] Korfage JA, Koolstra JH, Langenbach GE, Van Eijden TM. 2005 Fiber-type composition of the human jaw muscles—(part 1) origin and functional significance of fiber-type diversity. J. Dent. Res. **84**, 774-783. (10.1177/154405910508400901) 16109984

[99] Taylor AB, Holmes MA. 2021 Fiber-type phenotype of the jaw-closing muscles in *Gorilla gorilla, Pan troglodytes, *and* Pan paniscus*: a test of the frequent recruitment hypothesis. J. Hum. Evol. **151**, 102938. (10.1016/j.jhevol.2020.102938) 33493971

[100] Herron TJ, Korte FS, McDonald KS. 2001 Loaded shortening and power output in cardiac myocytes are dependent on myosin heavy chain isoform expression. Am. J. Physiol. Heart Circ. Physiol. **281**, H1217-H1222. (10.1152/ajpheart.2001.281.3.H1217) 11514290

[101] Sciote JJ, Kentish JC. 1996 Unloaded shortening velocities of rabbit masseter muscle fibres expressing skeletal or alpha-cardiac myosin heavy chains. J. Physiol. **492**, 659-667. (10.1113/jphysiol.1996.sp021335) 8734979 PMC1158889

[102] Qin H, Hsu MK, Morris BJ, Hoh JF. 2002 A distinct subclass of mammalian striated myosins: structure and molecular evolution of ‘superfast’ or masticatory myosin heavy chain. J. Mol. Evol. **55**, 544-552. (10.1007/s00239-002-2349-6) 12399928

[103] Rome L, Sosnicki A, Goble D. 1990 Maximum velocity of shortening of three fibre types from horse soleus muscle: implications for scaling with body size. J. Physiol. **431**, 173-185. (10.1113/jphysiol.1990.sp018325) 2100306 PMC1181769

[104] Andruchova O, Stephenson GM, Andruchov O, Stephenson DG, Galler S. 2006 Myosin heavy chain isoform composition and stretch activation kinetics in single fibres of *Xenopus laevis* iliofibularis muscle. J. Physiol. **574**, 307-317. (10.1113/jphysiol.2006.109926) 16644798 PMC1817808

[105] Pette D, Staron RS. 2000 Myosin isoforms, muscle fiber types, and transitions. Microsc. Res. Tech. **50**, 500-509. (10.1002/1097-0029(20000915)50:6<500::AID-JEMT7>3.0.CO;2-7) 10998639

[106] Staron RS, Pette D. 1993 The continuum of pure and hybrid myosin heavy chain-based fibre types in rat skeletal muscle. Histochemistry **100**, 149-153. (10.1007/BF00572901) 8244766

[107] Anapol F, Barry K. 1996 Fiber architecture of the extensors of the hindlimb in semiterrestrial and arboreal guenons. Am. J. Phys. Anthropol. **99**, 429-447. (10.1002/(SICI)1096-8644(199603)99:3<429::AID-AJPA5>3.0.CO;2-R) 8850183

[108] Anapol F, Gray JP. 2003 Fiber architecture of the intrinsic muscles of the shoulder and arm in semiterrestrial and arboreal guenons. Am. J. Phys. Anthropol. **122**, 51-65. (10.1002/ajpa.10269) 12923904

[109] Felder A, Ward SR, Lieber RL. 2005 Sarcomere length measurement permits high resolution normalization of muscle fiber length in architectural studies. J. Exp. Biol. **208**, 3275-3279. (10.1242/jeb.01763) 16109889

[110] Lieber RL. 2010 Skeletal muscle structure, function, and plasticity. Philadelphia, PA: Wolters-Kluwer.

[111] Méndez J. 1960 Density and composition of mammalian muscle. Metabolism **9**, 184-188.

[112] Kojima R, Medina MF, Jouffroy FK, Okada M. 2002 Effects of fixation and preservation conditions on immunohistochemical profiles of the skeletal muscle fibers in Japanese macaques. Z. Morphol. Anthropol. **83**, 315-324. (10.1127/zma/83/2002/315) 12050901

[113] Werner M, Chott A, Fabiano A, Battifora H. 2000 Effect of formalin tissue fixation and processing on immunohistochemistry. Am. J. Surg. Pathol. **24**, 1016-1019. (10.1097/00000478-200007000-00014) 10895825

[114] Huq E, Taylor AB, Su Z, Wall CE. 2018 Fiber type composition of epaxial muscles is geared toward facilitating rapid spinal extension in the leaper *Galago senegalensis*. Am. J. Phys. Anthropol. **166**, 95-106. (10.1002/ajpa.23405) 29318571 PMC5910278

[115] Schneider CA, Rasband WS, Eliceiri KW. 2012 NIH Image to ImageJ: 25 years of image analysis. Nat. Methods **9**, 671-675. (10.1038/nmeth.2089) 22930834 PMC5554542

[116] Galler S, Puchert E, Gohlsch B, Schmid D, Pette D. 2002 Kinetic properties of cardiac myosin heavy chain isoforms in rat. Pflüg. Arch. **445**, 218-223. (10.1007/s00424-002-0934-6) 12457242

[117] Kwa S, Weijs W, Juch P. 1995 Contraction characteristics and myosin heavy chain composition of rabbit masseter motor units. J. Neurophysiol. **73**, 538-549. (10.1152/jn.1995.73.2.538) 7539059

[118] Van Wessel T, Langenbach G, Korfage J, Brugman P, Kawai N, Tanaka E, Van Eijden T. 2005 Fibre-type composition of rabbit jaw muscles is related to their daily activity. Eur. J. Neurosci. **22**, 2783-2791. (10.1111/j.1460-9568.2005.04466.x) 16324112

[119] Perry JM, Hartstone-Rose A, Logan RL. 2011 The jaw adductor resultant and estimated bite force in primates. Anat. Res. Int. **112**, 929848. (10.1155/2011/929848) PMC3349241 22611496

[120] Weijs W, Hillen B. 1985 Cross-sectional areas and estimated intrinsic strength of the human jaw muscles. Acta Neerl. Morph. **23**, 267-274. 4096273

[121] Thomason J. 1991 Cranial strength in relation to estimated biting forces in some mammals. Can. J. Zool. **69**, 2326-2333. (10.1139/z91-327)

[122] Hoh JF, Kang LH, Sieber LG, Lim JH, Zhong WW. 2006 Myosin isoforms and fibre types in jaw-closing muscles of Australian marsupials. J. Comp. Physiol. **176**, 685-695. (10.1007/s00360-006-0091-x) 16773370

[123] Herring SW, Wineski LE. 1986 Development of the masseter muscle and oral behavior in the pig. J. Exp. Zool. **237**, 191-207. (10.1002/jez.1402370206) 3950565

[124] Vinyard CJ, Wall CE, Williams SH, Hylander WL. 2008 Patterns of variation across primates in jaw-muscle electromyography during mastication. Am. Zool. **48**, 294-311. (10.1093/icb/icn071) 21669792

[125] Wroe S, McHenry C, Thomason J. 2005 Bite club: comparative bite force in big biting mammals and the prediction of predatory behaviour in fossil taxa. Proc. R. Soc. B **272**, 619-625. (10.1098/rspb.2004.2986) PMC1564077 15817436

[126] Stienen G, Kiers J, Bottinelli R, Reggiani C. 1996 Myofibrillar ATPase activity in skinned human skeletal muscle fibres: fibre type and temperature dependence. J. Physiol. **493**, 299-307. (10.1113/jphysiol.1996.sp021384) 8782097 PMC1158918

[127] Zajac FE. 1989 Muscle and tendon: properties, models, scaling, and application to biomechanics and motor control. Crit. Rev. Biomed. Eng. **17**, 359-411. 2676342

[128] Holmes M, Taylor AB. 2021 Data from: The influence of jaw-muscle fibre-type phenotypes on estimating maximum muscle and bite forces in primates. *FigShare*. 10.1098/rsfs.2021.0009 PMC8361599 34938437

